# Supplementary material for: The interactome of intact mitochondria by cross-linking mass spectrometry provides evidence for coexisting respiratory supercomplexes
Source: Mol Cell Proteomics. 2017 Dec 8;17(2):216–32. doi: 10.1074/mcp.RA117.000470 (PMC5795388; doi:10.1074/mcp.RA117.000470)
Supplement: Supplemental Data [file supp_RA117.000470_133922_0_supp_23978_fzffwf.zip › spectra_annotation/mito_spectra_annotation/447-1-1-1-4-1.pdf]

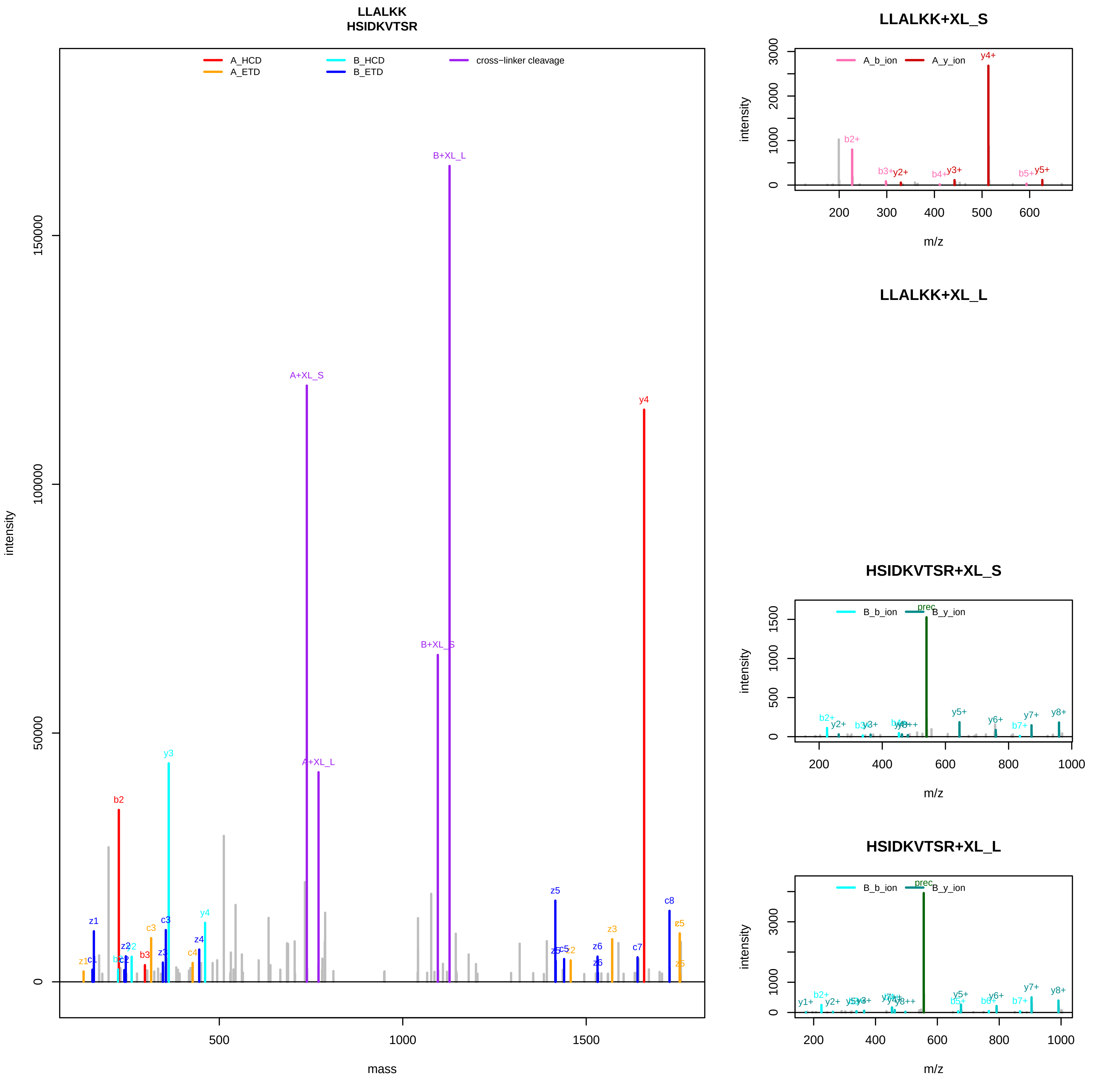

LLALKK
HSIDKVTSR
A_HCD
A_ETD
B_HCD
B_ETD
cross−linker cleavage
B+XL_L
A+XL_S
y4
B+XL_S
A+XL_L
y3
b2
c8
z5
c3
c7
z3
z6
y4
z1
intensity
mass
0
50000
100000
150000
500
1000
1500
LLALKK+XL_S
A_b_ion
A_y_ion
y4+
b2+
b3+
y2+
b4+
y3+
b5+
y5+
m/z
LLALKK+XL_L
HSIDKVTSR+XL_S
B_b_ion
B_y_ion
prec
y5+
y6+
y7+
y8+
b7+
HSIDKVTSR+XL_L
